# Bacterial colonization of the upper airways of children positive and negative for SARS-CoV-2 during the COVID-19 pandemic

**DOI:** 10.1186/s12879-022-07851-z

**Published:** 2022-11-17

**Authors:** Vincentia Rizke Ciptaningtyas, Rebriarina Hapsari, Endang Sri Lestari, Helmia Farida, Quirijn de Mast, Marinus Isaäk de Jonge

**Affiliations:** 1 Department of Microbiology, Faculty of Medicine, Universitas Diponegoro, Jl. Prof. H. Soedarto, SH, Tembalang, Semarang, 50275 Indonesia; 2 Diponegoro National Hospital, Semarang, Indonesia; 3 Department of Laboratory Medicine, Laboratory of Medical Immunology, Radboud Center for Infectious Diseases, Radboud Institute for Molecular Life Sciences, Radboud University Medical Center, Nijmegen, The Netherlands; 4 Department of Internal Medicine, Radboud Center for Infectious Diseases, Radboud University Medical Center, Nijmegen, The Netherlands

**Keywords:** Bacterial colonization, COVID-19 pandemic, Children, Indonesia

## Abstract

**Background:**

Our understanding of the influence of severe acute respiratory syndrome coronavirus 2 (SARS-CoV-2) infection on bacterial colonization in the children’s upper nasopharyngeal tract during the coronavirus infectious disease (COVID-19) pandemic is limited. This study aimed to determine whether there were any differences in bacterial colonization between asymptomatic children with or without a positive SARS-CoV-2 quantitative reverse transcriptase-polymerase chain reaction (RT-qPCR) results in the community setting.

**Methods:**

A cross-sectional community-based exploratory study was conducted from March to May 2021 in Semarang, Central Java Province, Indonesia. Using stored nasopharyngeal swabs collected from children under 18 years as a contact tracing program, we performed a real-time quantitative (qPCR) for the most important bacterial colonizing pathogens: *Streptococcus pneumoniae*, *Haemophilus influenzae*, *Staphylococcus aureus*, and *Klebsiella pneumoniae.*

**Results:**

Swabs from a total of 440 children were included in this study, of which 228 (51.8%) were RT-qPCR-confirmed SARS-CoV-2 positive. In the 440 children, colonization rates were highest for *H. influenzae* (61.4%), followed by *S. pneumoniae* (17.5%), *S. aureus* (12.0%), and *K. pneumoniae* (1.8%). The co-occurrence of both *S. pneumoniae* and *H. influenzae* in the upper respiratory tract was significantly associated with a SARS-CoV-2 negative RT-qPCR. In contrast, colonization with only *S. aureus* was more common in SARS-CoV-2-positive children.

**Conclusion:**

Overall, this exploratory study concludes that there is a significant difference in the bacterial nasopharyngeal colonization pattern between SARS-CoV-2 positive and negative in asymptomatic children in the community in Indonesia.

**Supplementary Information:**

The online version contains supplementary material available at 10.1186/s12879-022-07851-z.

## Background

The severe acute respiratory syndrome coronavirus 2 (SARS-CoV-2) is a highly transmissible and pathogenic coronavirus that first emerged in late 2019 and has since caused a pandemic of coronavirus disease 2019 (COVID-19), which poses a threat to human health and public safety globally [1]. A variety of symptoms, ranging from mild to severe respiratory failure, indicate the pathophysiology of SARS-CoV-2 infection in people. SARS-CoV-2 replicates primarily in the epithelial cells of the upper respiratory tract, from where it spreads and penetrates the lungs [2]. However, as the body of knowledge about the continuing pandemic grows, it seems that young children are less vulnerable to severe symptoms of SARS-CoV-2 infection [3].

Several studies have been conducted to determine the existence of SARS-CoV-2 co-infection with respiratory bacteria in the majority of adult patients who have contracted the virus. Gram-negative bacilli: *Acinetobacter baumannii*, *Klebsiella pneumoniae, Pseudomonas aeruginosa, Stenotrophomonas maltophilia* and *Staphyloccocus aureus* were commonly found as hospital-acquired superinfections*,* while *Streptococcus pneumoniae, Staphylococcus aureus,* and *Haemophilus influenzae* were found as community-acquired co-infection [4–6].

*S. pneumoniae, H. influenzae, S. aureus,* and *K. pneumoniae* are significant colonizers of children’s upper respiratory tracts that lead to lower respiratory tract infections, the leading cause of death in children under five years [7–10]. *S. pneumoniae* and *H. influenzae* are the most common bacterial causes of pneumonia, with *S. aureus* and *K. pneumoniae* associated with certain severe cases [11]. *K. pneumoniae,* along with other Gram-negative bacilli*,* is a more prevalent cause of pneumonia in Asia than in other regions worldwide [10]. By systematically searching the literature, we found that *K. pneumoniae* was the most frequently reported bacterial agent of lower respiratory tract infection in children under the age of five in Indonesia [12].

There is very little information about the association between the SARS-CoV-2 virus and bacterial colonization in asymptomatic children during public health measures during the COVID-19 pandemic. Therefore, this exploratory research aimed to determine whether there were any differences in *S. pneumoniae, H. influenzae, S. aureus*, and *K. pneumoniae* colonization between asymptomatic children with positive and negative SARS-CoV-2 RT-qPCR results in the community setting in Indonesia.

## Methods

### Study design and setting

A cross-sectional community-based exploratory study was conducted from March to May 2021 in Semarang, Central Java Province, Indonesia.

### Study population

The inclusion criteria in this study were the availability of nasopharyngeal swabs of children below the age of 18 years that were recently (less than two weeks before the visit) in contact with a confirmed COVID-19 patient from which swab samples were collected for contact tracing and were asymptomatic during the sample collection. We excluded swabs with an insufficient sample volume or incomplete personal data (date of birth, sex, national identification number). The Ethical Committee of the Faculty of Medicine Universitas Diponegoro approved the study protocol (No.64/EC/KEPK/FK-UNDIP/III/2021).

### Clinical data collection

We extracted age and sex data from the patient registry in the laboratory information system.

### Sample collection

The nasopharyngeal swabs were collected by trained nurses from Primary Health Centres (Puskesmas) in viral transport medium across Central Java and were sent to the Microbiology Laboratory, Faculty of Medicine, Universitas Diponegoro, Semarang, Indonesia. This laboratory is the designated reference laboratory for SARS-CoV-2 PCR testing under standardized clinical laboratory conditions.

In order to provide a reliable comparison between SARS-CoV-2 positive and negative swab samples, we selected all positive samples together with a similar number of randomly selected negative samples from the same day. If the SARS-CoV-2 positive sample was higher than the number of negative samples on a respective day, all available negative samples were selected on that particular day. All samples were aliquoted (1 ml) and stored in a − 80 °C freezer until further analysis.

### Quantitative real-time PCR (qPCR)

DNA for the positive controls was extracted from *S. pneumoniae* (ATCC® 49,619™), *S. aureus* (ATCC® 25,923™), *K. pneumoniae* (ATCC® 33,495™), and *H. influenzae* (ATCC® 49,247™) using PureLink™ Genomic DNA Mini Kit (cat.no.K182001, Invitrogen™, USA) based on the protocol for Gram-positive and Gram-negative bacteria. For real-time PCR standards, sequential dilution (tenfold) of the DNA spanning seven orders of magnitude starting at 10 ng/ µl was prepared in two sets on each plate to validate PCR efficiency across the sample.

Each sample was thawed, vortexed, and aliquoted (100 µl) into a microcentrifuge tube (cat.no 1210-SoS, SSIbio®, USA). The aliquoted samples were incubated at 95 °C for 15 min to lyse the bacteria and extract their DNA. Based on previous studies, four sets of primers (Table 1) based on highly conserved gene-specific for each bacterium were used [13–16]. The PCR was all performed using RealQ Plus 2 × Master Mix for Probe without ROX™ (cat.no.A313402, Ampliqon, Denmark) two-step PCR program in CFX Connect™ Real-Time PCR Detection System (Bio-Rad, USA). The PCR was done in a 25 µl reaction containing 1 µl of template DNA. Cycling conditions were 95 °C for 15 min, followed by 49 cycles of 95 °C for 15 s, and 20 s at 61 °C for *S. pneumoniae* and *K. pneumoniae*, 60 °C for *S. aureus*, and 55 °C for *H. influenzae*, respectively. Samples in which the quantification cycle (Cq) values < 40 were measured were considered positive.

**Table 1:** Primers and probes used for the qPCR assays to detect *S. pneumoniae*, *H. influenzae*, *S. aureus*, and *K. pneumoniae*

Organism (target gene)	Primer name	Nucleotide sequence of primers and probes
*S. pneumoniae (lytA)*	lytA-CDC-f	5ʹ-ACGCAATCTAGCAGATGAAGCA-3ʹ
	lytA-CDC-r	5ʹ-TCGTGCGTTTTAATTCCAGCT-3ʹ
	lytA-CDC-pr	5ʹ-FAM-GCCGAAAACGCTTGATACAG GGAG-3ʹ- BHQ1
*S. aureus (nuc)*	nucF	5ʹ-*GTTGCTTAGTGTTAACTTTAGTTGTA*-3ʹ
	nucR	5ʹ-*AATGTCGCAGGTTCTTTATGTAATTT*-3ʹ
	nucPr	5ʹ-FAM*-AAGTCTAAGTAGCTCAGCAAATGCA*-3ʹ- BHQ1
*K. pneumoniae (mdh)*	mdhF	5ʹ-CGGGCGTAGCGCGTAA-3ʹ
	mdhR	5ʹ-GATACCCGCATTCACATTAAACAG-3ʹ
	mdhP	5ʹ-FAM-CCCGGCATGGATCGTTCCGA-3ʹ-BHQ1
*H. influenzae (hpd)*	hpdF729	5ʹ-AGATTGGAAAGAAACACAAGAAAAAGA-3ʹ
	hpdR819	5ʹ-CACCATCGGCATATTTAACCACT-3ʹ
	hpdPbr762i	5ʹ-FAM-AAACATCCAATCGT-BHQ1-AATTATAGTTTACCCAATAACCC-3ʹ- C6

### Statistical analysis

We computed the Mann–Whitney U test to determine the statistical difference in the median age between those that tested positive and negative for COVID-19. Using Pearson’s chi-square tests or Fisher’s exact test, we compared age groups and sex between SARS-CoV-2 infected and uninfected children; bacterial species identified in children of different age groups, and co-colonization of bacterial species in children tested positive and negative for COVID-19. To determine the statistically significant differences in the mean rank of the Cq value among four bacteria, we used the Kruskal–Wallis H test. The effect size of the association between two nominal variables (bacteria-virus co-occurrence) was determined using Odds Ratio (95% confidence interval (CI)). All tests were performed on a two-tail hypothesis, and a *p* value below 0.05 was considered statistically significant. All data collected were analysed using SPSS® Version 26.0 (IBM Corp. Release 2019. IBM® SPSS® Statistics for Macintosh, Version 26.0. Armonk, NY).

## Results

### Demographic characteristic

A total of 440 samples from healthy and asymptomatic children were collected in this study. Two hundred twenty-eight (51.8%) children had SARS-CoV-2 positive RT-qPCR results. The majority were children aged twelve to seventeen (50.5%) and females (50.9%). The median (IQR) age of the children with positive or negative SARS-CoV-2 RT-qPCR results was 12 (7) and 11(8) years, respectively. A marginally significant association was found for SARS-CoV-2 positivity within the age group (*p* = 0.055), and no statistically significant association was found for SARS-CoV-2 positivity within gender (*p* = 0.44) (Table 2).

**Table 2:** Demographic characteristics of the study participants

	SARS-CoV-2 negative (*N* = 212) *n* (%)	SARS-CoV-2 positive (*N* = 228) *n* (%)	*p*
Age (median; IQR)	12; 7	11; 8	0.023*
Age groups (years)			0.055^+^
0–5	49 (23.1)	35 (15.4)	
6–11	67 (31.6)	67 (29.4)	
12–17	96 (45.3)	126 (55.3)	
Sex			
Male	100 (47.2)	116 (50.9)	0.44
Female	112 (52.8)	112 (49.1)	

*Variables analysed using the Mann–Whitney U test, *p* < 0.05

^+^Variables analysed using Pearson’s chi-square test, marginally significant

### Bacterial colonization in the upper respiratory tract

**Fig. 1:**
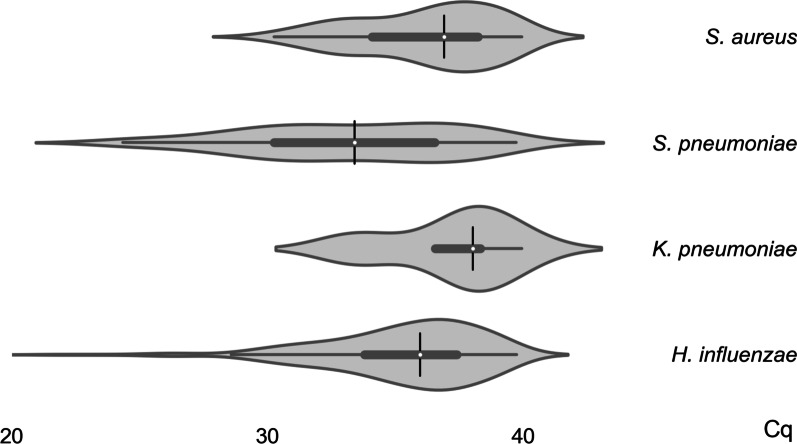
Cq value distribution among isolated bacteria. Violin plot representation of Cq value of *S. aureus, S. pneumoniae, K. pneumoniae*, and *H. influenzae* from positive samples (Cq values < 40)

The Cq values from all samples, indicative of the density of bacterial colonization, were determined for *S. aureus*, *S. pneumoniae*, *K. pneumoniae*, and *H. influenzae* (Fig. 1). *S. pneumoniae* had the lowest Cq value median (33.4), followed by *H. influenzae* (35.9), *S. aureus* (36.9), and *K. pneumoniae* (38.0) (*p* < 0.001). *H. influenzae* had the highest colonization rate (61.4%), followed by *S. pneumoniae* (17.5%), *S. aureus* (12.0%), and *K. pneumoniae* (1.8%) (Table 3). There was a significant association between age and overall colonization with *S. aureus* (*p* = 0.003) and *S. pneumoniae* (*p* = 0.003), with the highest colonization rates in 6–11 years (Table 3).

**Table 3:** Comparison of SARS-CoV-2 and bacterial species identified in children of different age groups

	Age 0–5 years (*N* = 84) *n* (%)	Age 6–11 years (*N* = 134) *n* (%)	Age 12–17 years (*N* = 222) *n* (%)	*p*
SARS-CoV-2	35 (15.4)	67 (29.4)	126 (55.3)	0.055^+^
*S. aureus*	7 (13.2)	27 (50.9)	19 (35.8)	0.003*
*S. pneumoniae*	12 (15.6)	36 (46.8)	29 (37.7)	0.003*
*K. pneumoniae*	2 (25.0)	2 (25.0)	4 (50.0)	0.80†
*H. influenzae*	53 (19.6)	83 (30.7)	134 (49.6)	0.89

The number for each bacterial species represents overall detection (regardless of the bacteria as a single colonizer or in the co-colonization state with other bacteria)

^+^Variables analysed using Pearson’s chi-square test, marginally significant

*Variables analysed using Pearson’s chi-square test, *p* < 0.05

†Variable analysed using Fisher’s exact test

### Co-colonization of bacterial species in the upper respiratory tract

Table 4 summarizes the colonization prevalence in children positive or negative for SARS-CoV-2. In most nasopharyngeal swab samples, only a single species was detected (Table 4). Although *H. influenzae* had the highest detection rate in children, those who were only colonized with *S. aureus* had higher odds of being SARS-CoV-2 positive (OR 3.9, 95% CI 1.1 to 13.9). In this population, co-occurrence of *S. pneumoniae* and *H. influenzae* dominates (61.5%) the other two bacterial species combinations. More importantly, this specific combination was significantly associated with SARS-CoV-2 negative RT-qPCR results (OR 0.5, 95% CI 0.3 to 0.9).

**Table 4:** Co-colonization of bacterial species in SARS-CoV-2 infected and uninfected children

	SARS-CoV-2 negative (*N* = 212) *n* (%)	SARS-CoV-2 positive (*N* = 228) *n* (%)	Odd ratio (95% CI)
No colonization	70 (33.0)	70 (30.7)	0.9 (0.6 to 1.3)
Single bacterial species	90 (42.5)	108 (47.4)	
* S. aureus*	3 (3.3)	12 (11.1)	**3.8** (1.1 to 13.9)*
* S. pneumoniae*	4 (4.4)	7 (6.5)	1.6 (0.5 to 5.7)
* K. pneumoniae*	2 (2.2)	0 (0.0)	NA
* H. influenzae*	81 (90.0)	89 (82.4)	1.0 (0.7 to 1.5)
Two bacterial species	50 (23.6)	46 (20.2)	
* S. aureus* and *S. pneumoniae*	0 (0.0)	2 (4.3)	NA
* S. aureus* and *H. influenzae*	10 (20.0)	20 (43.5)	1.9 (0.9 to 4.3)
* S. pneumoniae* and *H. influenzae*	37 (74.0)	22 (47.8)	**0.5** (0.3 to 0.9)*
* K. pneumoniae* and *H. influenzae*	3 (6.0)	2 (4.3)	0.6 (0.1 to 3.7)
Three bacterial species	2 (0.9)	4 (1.8)	
* S. aureus* and *S. pneumoniae* and *H. influenzae*	2 (100.0)	3 (75.0)	1.4 (0.2 to 8.5)
* S. aureus* and *K. pneumoniae* and *H. influenzae*	0 (0.0)	1 (25.0)	NA

*NA*  not applicable, *CI*  confidence interval

*Variables analysed using Pearson’s chi-square test, *p* < 0.05

## Discussion

This study was conducted during the second wave of the COVID-19 pandemic, when the delta variant was most prevalent [17] in Indonesia, to determine any differences in the colonization patterns of *S. pneumoniae*, *H. influenzae*, *S. aureus*, and *K. pneumoniae* between SARS-CoV-2 positive and negative children. In this study, it was found that *H. influenzae* had the highest colonization rate. Interestingly, co-colonization of *S. pneumoniae* and *H. influenzae* in the nasopharynx was associated with the absence of SARS-CoV-2 infection, while children only colonized with *S. aureus* had higher odds of having SARS-CoV-2 positive results. These results suggest a difference in bacterial colonization patterns between SARS-CoV-2 RT-qPCR positive and negative in asymptomatic children.

In this study, we found that from all 440 children included, *H. influenzae* had the highest colonization rate, followed by *S. pneumoniae*, *S. aureus*, and *K. pneumoniae*. This result differs from pre-COVID-19 studies, which found the highest colonization rates for *S. pneumoniae*, followed by *H. influenzae* and *S. aureus* [12]. A pre-COVID-19 study among HIV-infected children in Jakarta and healthy children in three provinces in Indonesia (West Java, West Nusa Tenggara, West Sumatra) showed 18% and 27,5% *H. influenzae* carrier rates [18, 19]. Compared to other Southeast Asia countries, the colonization rate of *H. influenzae* in our study is also considerably higher. A study in Malaysia showed 14,3% and 4,9% *H. influenzae* carrier rates in healthy unvaccinated and vaccinated children, respectively [20]. The Indonesian ministry of health started to include the Hib vaccination in the national vaccination program in 2013; this might have caused the higher carrier rate of *H. influenzae* in this study since most of our participants were older than 5 years old.

While an earlier study suggested that bacterial co-infection with *H. influenzae*, *S. pneumoniae*, increased the risk of morbidity and mortality in patients hospitalized due to community-acquired pneumoniae and SARS-CoV-2 infection [21, 22], we found that this specific combination of pathogenic bacterial species was associated with reduced occurrence of SARS-CoV-2 infection in children. Co-occurrence of *H. influenzae* and *S. pneumoniae,* leading to increased density of *H. influenzae* in the presence of *S. pneumoniae* in a neonatal rat model, was previously shown and suggested synergy between these species [23]. A possible explanation is a nutritional dependency in which *S. pneumoniae* provides nutrients to *H. influenzae*, as observed for *S. aureus* and *H. influenzae,* described as the ‘satellite phenomenon’ [23]. It is tempting to speculate that this synergistic interaction in the nasopharynx elicits mucosal immune responses leading to inhibition of infection with SARS-CoV-2, although a causal link has not been demonstrated in this study.

The association of SARS-CoV-2 infection and *S. aureus* colonization, as found in this study, is in line with other studies [22] showing that although bacterial co-infection in COVID-19 patients was low, *S. aureus* was the most commonly found species. Regarding *K. pneumoniae,* we found 1.8% carriage in all samples. This result is lower than a study held eight years ago in Indonesia that found 7% of *K. pneumoniae* carriage in children under five years of age [24]. However, as proposed by Farida and colleagues, exposure to unsafe food and water was likely the culprit of the previously high carrier rate [24]; during mobility reduction and school closure, children were less exposed to street food, thus reducing the change of *K. pneumoniae* transmission in the pandemic era.

We acknowledge some limitations in this study. First, we had no access to information on the history of the children’s vaccination and current antibiotic use; thus, we cannot compare the bacterial colonization among children who have received a vaccination and have used antibiotics and those who have not. Second, there is no exact data related to when the children were exposed to SARS-CoV-2 infection. Despite these limitations, to our knowledge, this is the first study to examine the differences in bacterial colonization between SARS-CoV-2 RT-qPCR positive and negative in asymptomatic children. Further studies using 16 s rRNA sequencing and metagenomics in both symptomatic and asymptomatic children would improve our understanding of global changes in the microbiome of the upper respiratory as a consequence of SARS-CoV-2 infection.

## Conclusion

This explorative study shows differences in the bacterial nasopharyngeal colonization pattern between asymptomatic SARS-CoV-2 positive and negative children during public health measures in the community in Indonesia. Differences in the colonization pattern might influence the epidemiology of bacterial respiratory tract infections in children, as well as modulate the risk for SARS-CoV-2 infection.

## Supplementary Information

**Additional file 1.** PCR Results.

## Data Availability

All data generated or analysed during this study are included in this published article and its supplementary information files (Additional file 1).
